# Clinical, Immunological, and Molecular Variability of RAG Deficiency: A Retrospective Analysis of 22 RAG Patients

**DOI:** 10.1007/s10875-021-01130-3

**Published:** 2021-10-18

**Authors:** Cristina Cifaldi, Beatrice Rivalta, Donato Amodio, Algeri Mattia, Lucia Pacillo, Silvia Di Cesare, Maria Chiriaco, Giorgiana Madalina Ursu, Nicola Cotugno, Carmela Giancotta, Emma C. Manno, Veronica Santilli, Paola Zangari, Galaverna Federica, Giuseppe Palumbo, Pietro Merli, Paolo Palma, Paolo Rossi, Gigliola Di Matteo, Franco Locatelli, Andrea Finocchi, Caterina Cancrini

**Affiliations:** 1 Academic Department of Pediatrics (DPUO), Immune and Infectious Diseases Division, Research Unit of Primary Immunodeficiencies, Bambino Gesù Children’s Hospital, IRCCS, 00165 Rome, Italy; 2 Chair of Pediatrics, Department of Systems Medicine, University of Rome “Tor Vergata”, via Montpellier, 1, 00133 Rome, Italy; 3 Academic Department of Pediatrics (DPUO), Research Unit of Clinical Immunology and Vaccinology, Bambino Gesù Children’s Hospital, IRCCS, 00165 Rome, Italy; 4 Department of Pediatric Hemato-Oncology and Cell and Gene Therapy, Scientific Institute for Research and Healthcare, Bambino Gesù Children’s Hospital, IRCCS, 00165 Rome, Italy; 5 Department of Pediatrics, Sapienza, University of Rome, Rome, Italy

**Keywords:** RAG deficiency, RAG1/RAG2, Hypomorphic mutation, CID phenotypes, Cytopenia

## Abstract

**Purpose:**

We described clinical, immunological, and molecular characterization within a cohort of 22 RAG patients focused on the possible correlation between clinical and genetic data.

**Methods:**

Immunological and genetic features were investigated by multiparametric flow cytometry and by Sanger or next generation sequencing (NGS) as appropriate.

**Results:**

Patients represented a broad spectrum of RAG deficiencies: SCID, OS, LS/AS, and CID. Three novel mutations in *RAG1* gene and one in *RAG2* were reported. The primary symptom at presentation was infections (81.8%). Infections and autoimmunity occurred together in the majority of cases (63.6%). Fifteen out of 22 (68.2%) patients presented autoimmune or inflammatory manifestations. Five patients experienced severe autoimmune cytopenia refractory to different lines of therapy. Total lymphocytes count was reduced or almost lacking in SCID group and higher in OS patients. B lymphocytes were variably detected in LS/AS and CID groups. Eighteen patients underwent HSCT permitting definitive control of autoimmune/hyperinflammatory manifestations in twelve of them (80%).

**Conclusion:**

We reinforce the notion that different clinical phenotype can be found in patients with identical mutations even within the same family. Infections may influence genotype–phenotype correlation and function as trigger for immune dysregulation or autoimmune manifestations. Severe and early autoimmune refractory cytopenia is frequent and could be the first symptom of onset. Prompt recognition of RAG deficiency in patients with early onset of autoimmune/hyperinflammatory manifestations could contribute to the choice of a timely and specific treatment preventing the onset of other complications.

**Supplementary Information:**

The online version contains supplementary material available at 10.1007/s10875-021-01130-3.

## Introduction

Systematic rearrangement of antigen receptor genes via V(D)J recombination is essential for maturation of progenitor lymphocytes, the genesis of immunoglobulin and T cell receptor (TCR), and production of a broad repertoire of antigen-specific T and B cells [1–6]. Complete RAGs deficiency has been known to cause severe combined immunodeficiency (SCID) phenotype with lack of T and B cells (T-B-NK+ SCID) [7], life-threatening infections, and failure to thrive in early infancy. Today, RAGs diseases are associated to an expanding broad spectrum of phenotypes ranging from SCID, Omenn syndrome (OS) [8–13], “leaky” or “atypical” SCID, (LS/AS) whose peculiarities reside in varying numbers of oligoclonal T and B cells, and in some cases a predominance of γδ+ T cells (γδ AS) and autoimmune cytopenias [14]. Hypomorphic mutations allowing a residual RAG protein function may lead to a later onset of different diseases and a risk of delayed diagnosis. These forms can be characterized by diffuse granulomatous disease and/or autoimmunity (CID-G/AI) and an altered response to severe Herpesviridae infections (in particular EBV) [15–17]. Otherwise, common variable immunodeficiency (CVID), idiopathic CD4+ T cell lymphopenia (ICL) [18], IgA deficiency, and hyper-IgM syndrome have been also reported [19–21].

Although infections are the predominant presenting features in RAG deficiency patients, autoimmune manifestations including cytopenia, autoimmune hepatitis, myopathy, and nephrotic syndrome [17, 22, 23] should be considered associated manifestations. In addition, patients may develop inflammation and immune dysregulation symptoms as dermatitis or lymphoproliferation due to hyperinflammation and altered lymphocyte homeostasis [30].

This autoimmunity has been linked not only to checkpoint breaks in both T and B cell tolerance but also to other mechanisms which may play an additional role in sustaining autoimmune pathology [24].

Herein, we report the clinical and immunological phenotype together with molecular characterization of 22 RAG patients referred to our center presenting with a broad spectrum of symptoms including autoimmune and/or hyperinflammatory manifestations.

## Methods

### Patients

A total of 22 patients from 18 different families were assessed between 2009 and 2020 at the Bambino Gesù Children’s Hospital. Patients were classified into four groups (SCID, OS, LS/AS, CID) based on clinical presentation, immunological data, on the criteria published by the Primary Immune Deficiency Treatment Consortium (PIDTC) [25], ESID 2019 [26], and other groups [24, 27, 28].

- SCID: at least one of: invasive bacterial, viral, or fungal/opportunistic infection; persistent diarrhoea and failure to thrive; affected family member AND manifestation in the first year of life AND two of 4 T cell criteria fulfilled: absence or very low number of T cells (CD3 T cells < 300/microliter), reduced naive CD4 and/or CD8 T cells; elevated γ/δ T cells; no or very low T cell function by response to mitogen or TCR stimulation; AND T cells maternal engraftment excluded AND HIV excluded.
- OS: Desquamating erythroderma in the first year of life AND one of the following: lymphoproliferation, hepatomegaly splenomegaly; failure to thrive; chronic diarrhea; recurrent pneumonia AND eosinophilia or elevated IgE AND T-cell deficiency (detectable CD3 T cells, ≥ 300/microliter, low naïve cells, reduced proliferation, oligoclonality) AND maternal engraftment excluded AND HIV excluded.
- LS/AS: Mutation in a SCID-causing gene **AND** Absence of characteristic SCID-associated infections (PCP, symptomatic CMV, persistent respiratory or gastrointestinal virus infection) in the first year of life **AND** does not fulfill criteria for Omenn syndrome. Reduced number of CD3 T cells (for age up to 2 years < 1000/microliter; for > 2 years up to 4 years < 800/microliter, for > 4 years < 600/microliter) AND Absence of maternal engraftment AND < 30% of lower limit of normal T cell function (as measured by response to PHA) **AND** Presence of activated, oligoclonal, and autologous T cells not associated with typical features of OS. Expansion of γδ T cells upon cytomegalovirus (CMV) infection or Epstein-Barr virus (EBV)-driven lymphoproliferative disease and autoimmune cytopenia could be associated.
- CID: at least one of: severe infection (especially VZV, CMV, EBV, HPV, and molluscum); one manifestation of immune dysregulation (autoimmunity, IBD, severe eczema, lymphoproliferation, granuloma); malignancy **AND** age ≥ 2 years **AND** affected family member AND 2 of 4 T cell criteria fulfilled: reduced CD3 or CD4 or CD8 T cells (using age-related reference values); reduced naïve CD4 and/or CD8 T cells; elevated g/d T cells; reduced proliferation to mitogen or TCR stimulation.

The clinical and immunological characterization is summarized in Tables 1 and 2.

**Table 1:** Clinical features of RAG patients

ID/Gender	Final clinical diagnosis	Gene/mutation	Age of manifestation	Age at genetic diagnosis	Infective complications	Autoimmune complications	Other autoimmune or hyperinflammatory complications	Autoantibody	Treatment	Long-term follow-up	Ref.
PID-1 F	SCID	*RAG2*	5 months	6 months	Long lasting gastroenteritis (adenovirus)				HSCT	Good	[50]
		(a) c.1A > G; p.M1V (b) c.1403_1406del; p.H468RfsTer16									
PID-2 M	SCID	*RAG1*	2 months	3 months	Chronic CMV, retinitis (CMV), bronchiolitis/pneumonia (respiratory syncytial virus, adenovirus)				HSCT	Good	[50]
		(a1) c.1681C > T; p.R561C (a2) c.1815G > C; p.M605I (b) c.2780 T > C; p.F927S									
PID-3 F	SCID	*RAG1*	6 months	6 months	Pneumonia				HSCT	Good	[50]
		(a) c.1229G > A; p.R410Q (b) c.1863delG; p.A622QfsTer9									
PID-4 M	SCID	*RAG1*	4 months	7 months	Pneumonia, long-lasting diarrhea (rotavirus, adenovirus)				HSCT	Good	[50]
		(a) c.987delC; p.S330LfsTer15									
PID-5 F	SCID	*RAG1*	3 months	7,5 months	Otitis (Pseudomonas aeruginosa), urinary infection, bronchiolitis		Dermatitis		HSCT	Good	
		(a) c.1228C > T; p.R410W (b) c.2780 T > C; p.F927S									
PID-6 F	SCID	*RAG1*	Birth	Prenatal diagnosis					HSCT	Good	
		(a) c.1871G > A; p.R624 (b) c.1213A > G; p.R405G									
PID-7 F	SCID	*RAG1*	7 months	7 months					HSCT	Good	
		(a) c.1361 T > A p. L454Q									
PID-8 M	SCID	*RAG1*	25 days	1 months	Chronic HHV6, long-lasting diarrhea (norovirus), pneumonia,		Dermatitis, splenomegaly		HSCT	Good	
		(a) c. 1210 C > T; p.R404W									
PID-9 M	OS	*RAG1*	2 months	4 months	Chronic CMV		Dermatitis (eritrodermia)		HSCT	Good	[50]
		(a) c.1682G > A; p.R561H (b) c.1871G > A; p.R624H									
PID-10 F	OS	*RAG1*	2,5 months	3 months	Pneumonia, sepsis		Dermatitis (eritrodermia)		HSCT	Good	
		(a) c.351delT; p.Phe118LeufsTer21 (b) c.1577 T > G; p.L526R									
PID-11 M	OS	*RAG1*	Birth	1 month	Pneumocystosis, pneumonia (flavobacterium meningosepticum)	ITP	Dermatitis (eritrodermia), tubule interstitial nephritis		IVIG, steroids, HSCT	Good	[50]
		(a) c.1870C > T; p.R624C (b) c.2521C > T; p.R841W									
PID-12 M	OS	*RAG2*	5 months	6 months	Pneumonia, sepsis, urinary tract infections		Dermatitis (eritrodermia), alopecia, splenomegaly		HSCT	Good	
		(a) c.281A > G; p.H94R (b) c.1090_1093delAACA; p.N364VfsTer79									
PID-13 M	OS	*RAG1*	3 months	4 months	Purulent otitis, necrotizing fasciitis, cerebral abscess, sepsis		Dermatitis (eritrodermia)			Passed away before HSCT for multiorgan failure	
		(a) c.519delT; p.E174Sfs*27									
PID-14 F	OS	*RAG1*	Birth	Prenatal diagnosis			Dermatitis (eritrodermia)		HSCT	Good	
		(a) c.1870C > T; p.R624C (b) c.2521C > T; p.R841W									
PID-15 M	AS	*RAG2*	5 months	6 months			Dermatitis, lymphadenopathy		HSCT		[50]
		(a) c.685C > T; p.R229W									
PID-16 M	AS	*RAG1*	8 months	1,2 years	Long-lasting diarrhea (adenovirus)		Dermatitis (eritrodermia), tubulointerstitial nephritis		HSCT	Good	[50]
		(a) c.2521C > T; p.R841W									
PID-17 M	AS	*RAG1*	9,2 months	1,1 years	Chronic CMV, pneumonia, sepsis (*P. aeruginosa*), BCG infection	Miller-Fisher syndrome (CMV-related), AIHA		Coombs, antiplatelets Ab, INFα Ab, INFω Ab	IVIG, steroid, rituximab, plasmapheresis, HSCT	Good	[27]
		(a) c.256_257del; p.K86VfsTer33									
PID-18 F	AS	*RAG1*	1,8 years	1,9 years	Chronic CMV, long lasting gastroenteritis (adenovirus)				HSCT	Good	
		(a) c.1767C > G; p.Y589X									
PID-19 F	CID	*RAG1*	3 years	3,4 years	Chronic HHV-6, CMV, EBV, neutropenia, pneumonia	AIHA		Coombs	IVIG, steroid, rituximab, HSCT	Passed away before for invasive pulmonary aspergillosis	[50]
		(a) c. 1871G > A; p.R624H (b) c.1213A > G; p.R405G									
PID-20 M	CID	*RAG1*	1 years	2 years	Chronic CMV, EBV, long-lasting diarrhea (rotavirus), pneumonia (haemophilus influenzae, bocavirus), sepsis	ITP, AIHA	Splenomegaly	ANA Ab, antiphospholipid Ab, antiplatelets Ab, coombs	IVIG, steroid, rituximab, plasmapheresis	Passed away before HSCT for multiorgan failure	[50]
		(a) c.2521C > T; p.R841W									
PID-21 M	CID	*RAG1*	6 years	11 years	Chronic EBV, severe chicken POX, pneumonia					Good	[39]
		(a) c.1871G > A; p.R624H (b) c.2182 T > C; p.Y728H									
PID-22 F	CID	*RAG1*	3 years	10 years	Otitis, pneumonia, chronic HHV6	AIHA, autoimmune thyroiditis	Vitiligo	Coombs, thyroglobulin ab	IVIG, steroid, rituximab, mycophenolic acid, rapamycin	Still admitted in severe clinical state	
		(a) c.2095C > T; p.R699W (b) c.2408A > G; p.N803S									

*SCID* severe combined immunodeficiency, *OS* Omenn syndrome, *AS* atypical SCID, *LS* Leaky/SCID, *CID* combined immunodeficiency, *EBV* Epstein-Barr virus, *CMV* cytomegalovirus, *HHV-6* human herpesvirus 6, *AIHA* autoimmune hemolytic anemia, *ITP* immune thrombocytopenia, *IFN* interferon, *ANA* antinuclear antibody, *HSCT* hematopoietic stem cell transplantation

**Table 2:** Immune phenotype of RAG cohort

ID/ disease	Eosinophils (cells/uL)	Lymphocytes (cells/uL)	CD3 + (%)	CD3 + (cells/uL)	CD4 + (%)	CD4 + (cells/uL)	CD8 + (%)	CD8 + (cells/uL)	CD19 + (%)	CD19 + (cells/uL)	CD16 + CD56 + (%)	CD16 + CD56 + (cells/uL)
PID-1 SCID	350	580	8	46.4	1.5	8.7	6.1	35.3	0.3	1.74	91	527.8
PID-2 SCID	20	290	0.2	0.58	0.1	0.29	0.1	0.29	0.1	0.29	96.4	279.5
PID-3 SCID	30	220	14.5	31.9	10.8	23.76	3.7	8.14	0	0	81.8	179.9
PID-4 SCID	30	350	n.a	n.a	n.a	n.a	n.a	n.a	n.a	n.a	n.a	n.a
PID-5 SCID	3670	1400	8.1	113.4	5.9	82.6	2.2	30.8	0.144	2.02	87.1	1219.4
PID-6 SCID	130	560	2.4	13.44	1.4	7.84	0.6	3.36	3.4	19.04	93.3	522.4
PID-7 SCID	770	310	64	198.4	28.7	88.97	19.2	59.52	0	0	32.4	100.4
PID-8 SCID	200	1292	32.1	414.73	8.9	114.9	20.8	268.7	0	0	66.9	864.3
PID-9 OS	7900	12.170	69.8	8494.6	38.9	4734.1	30.9	3761	0	0	28.6	3480.6
PID-10 OS	540	10.830	91	9855.3	10	1083	77	8339	0	0	4	433.2
PID-11 OS	5700	3120	64.7	2018.6	34	1060.8	26.3	820.5	0.2	6.24	32.6	1017.1
PID-12 OS	5910	2800	84	2352	82	2296	3	84	1	28	2.74	76.72
PID-13 OS	1850	830	80.5	668.15	71.6	594.28	4.4	36.52	0.6	4.98	16.3	135.29
PID-14 OS	7420	2800	88.9	2489.2	82.8	2318.4	6.1	170.8	0.2	5.6	10.1	282.8
PID-15 LS/AS	380	270	54.4	146.8	49.9	134.7	4.7	12.6	0	0	42.8	115.5
PID-16 LS/AS	150	550	50.1	275.55	11.6	63.8	1.1	6.05	6.4	35.2	35.2	193.6
PID-17 LS/AS	40	2760	50.2	1385.5	2.8	77.2	32.3	891.4	37.9	1046	10.4	287.0
PID-18 LS/AS	30	450	n.a	n.a	n.a	n.a	n.a	n.a	n.a	n.a	n.a	n.a
PID-19 CID	0	1950	58.5	1140.75	28.3	551.8	18.4	358.8	1.8	35.1	39.1	762.4
PID-20 CID	50	2420	11	266.2	10.1	244.4	0.5	12.1	27.3	661	52	1258.4
PID-21 CID	130	880	75.4	663.5	44.9	395.1	20.4	179.5	0.8	7.04	21.9	192.7
PID-22 CID	72	1500	48.4	726	31.7	475.5	13.4	201	21.7	325.5	27.8	417

ID/disease	CD31 + (%)	CD31 + (cells/uL)	CD3 + αβ (%)	CD3 + γδ (%l)	CD4 + CD45RA + (%)	CD4 + CD45RA + (cells/uL)	CD4 + CD45RO + (%)	CD4 + CD45RAO + (cells/uL)	IgG (mg/dL)	IgM (mg/ dL)	IgA (mg/dL)	IgE (IU/mL)
PID-1 SCID	n.a	n.a	n.a	n.a	n.a	n.a	n.a	n.a	186 **	10	8	10
PID-2 SCID	n.a	n.a	n.a	n.a	n.a	n.a	n.a	n.a	802 *	10	5	< 5
PID-3 SCID	n.a	n.a	n.a	n.a	n.a	n.a	n.a	n.a	1699 *	10	13	< 5
PID-4 SCID	n.a	n.a	n.a	n.a	n.a	n.a	n.a	n.a	348 **	10	5	< 5
PID-5 SCID	n.a	n.a	n.a	n.a	n.a	n.a	n.a	n.a	n.a	n.a	n.a	n.a
PID-6 SCID	n.a	n.a	98.3	1.3	n.a	n.a	n.a	n.a	809 *	20	5	19.3
PID-7 SCID	14.5	1.1	n.a	n.a	n.a	n.a	n.a	n.a	8.46	0.05	0.04	0
PID-8 SCID	n.a	n.a	n.a	n.a	n.a	n.a	n.a	n.a	432	17.3	4	84
PID-9 OS	n.a	n.a	n.a	n.a	0.4	18.94	38.5	1822	100	10	5	8629
PID-10 OS	n.a	n.a	n.a	n.a	n.a	n.a	n.a	n.a	240 **	24	10	> 5000
PID-11 OS	n.a	n.a	84	15.7	0.3	3.18	33.7	357.49	268 **	10	5	15,500
PID-12 OS	n.a	n.a	n.a	n.a	n.a	n.a	n.a	n.a	183 **	10	5	7074
PID-13 OS	1.4	8.3	n.a	n.a	1.4	8.32	70.2	417.18	n.a	n.a	n.a	n.a
PID-14 OS	n.a	n.a	94.7	3.9	0.8	18.5	82	1901.1	825	46	40	2251
PID-15 LS/AS	n.a	n.a	n.a	n.a	1.6	2.16	31.5	42.44	864	10	5	n.a
PID-16 LS/AS	0.06	0.03	25.4	70.7	0.2	0.13	11.4	7.27	538	54	146	< 5
PID-17 LS/AS	50.1	38.7	27.8	70.4	0.1	0.08	2.7	2.9	1981	209	137	5.6
PID-18 LS/AS	n.a	n.a	n.a	n.a	n.a	n.a	n.a	n.a	910 *	10	25	10
PID-19 CID	n.a	n.a	2.5	7.5	0.3	1.66	28	154.5	10	10	10	< 5
PID-20 CID	0.07	0.2	95.3	3	0.29	1.66	9.8	24	1142	157	88	104
PID-21 CID	2.5	9.9	88.5	10.1	4	15.8	40.9	161.6	8	10	5	< 5
PID-22 CID	0.7	1.82	93	6	3.1	14.7	28.6	136	858	196	76	< 5

PID-4 and PID-18 no immune phenotype data available

PID-5 and PID-8 maternal engraftment

^*^On IVIG

Pre IVIG IgG value of PID-14

^**^Maternal origin

*n.a.* Not available

Two patients (PID-17 and PID-21) have been enrolled in the Profound CID study (pCID) (DRKS00000497), fulfilling the study inclusion criteria.

### Multiparametric Flow Cytometric Analysis

All flow cytometric analyses were performed on ethylenediamine tetraacetic acid (EDTA) blood samples within 24 h of venipuncture. After red blood cell lysis with ammonium chloride, the lymphocytes were incubated with the appropriate antibody cocktail for 30 min at 4 °C, washed with PBS, and suspended in PBS. At least 50,000 events in the lymphocyte live gate were acquired for each sample. Samples were acquired on FACSCANTO II (BD Biosciences, San Diego, CA, USA) and analyzed with FlowJo software (Tree Star Inc, version 8.8.6, Ashland, Ore).

### Genetic Analysis

Ion Torrent Gene Target Library Preparation and NGS Sequencing were performed according to manufacturer’s instructions (Thermo Fisher Scientific).

Next generation sequencing (Ion Torrent) and sequencing: genomic DNA is isolated from peripheral blood of patients using standard protocols (QIAamp DNA Blood kit by QIAGEN GmbH, Hilden, Germany). PCR reactions were carried out using GoTaq DNA polymerase by standard methods (Promega, Madison, WI). Direct sequencing was performed using the BigDye Terminator v3.1 Cycle Sequencing Kit (Applied Biosystems, Foster City, CA) and analyzed on an ABI PRISM 3130 and 310 automated sequencers (Applied Biosystems).

Sanger sequencing for all mutations and parents’ carrier status was performed.

### Ion Torrent Bioinformatics Analysis

Mapping and variant calling were performed using the Ion Torrent suite software v3.6. Sequencing reads were aligned against the USC hg19 reference genome using the program distributed within the Torrent mapping Alignment Program (TMAP) map4 algorithm (Thermo Fisher; https://github.com/iontorrent/TS). The aligned reads were processed for variant calling by using the Torrent Suite Variant Caller TVC program; variants found in Variant Calling Format (VCF) file were annotated using ANNOVAR. The called variants with minimum coverage of ×20, standard Mapping Quality, and Base Phred Quality were examined on Integrative Genome Viewer (IGV) and BIOMART. After applying filtering criteria, all nonsense, frameshift, and canonical splice site variants were evaluated to determine their potential pathogenicity.

### Statistical Analysis

Data were analyzed with Graph Pad Prism, version 6.2 (Graph Pad Software, La Jolla, CA). *p* < 0.05 and **p < 0.005 were considered significant.

## Results

### Clinical Phenotype and Genetic Characterization of RAG Cohort

We report clinical, immunological, and molecular characterization within a cohort of 22 RAG patients diagnosed between 2009 and 2020.

**Fig. 1:**
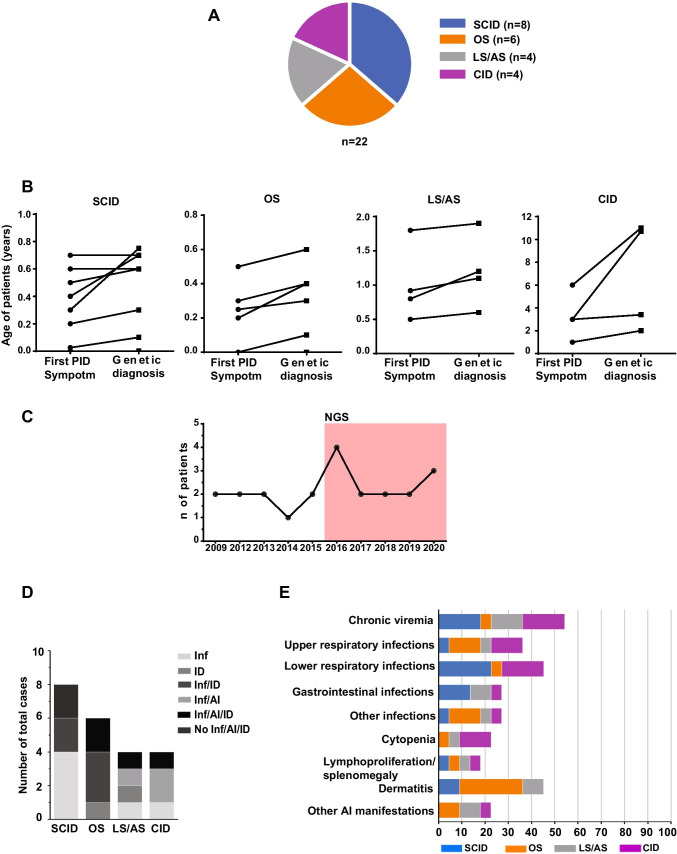
**a** RAG cohort. Clinical diagnosis of RAG patients for the four main categories (*n* = 22). **b** Difference in timing for first symptom of immunodeficiency and genetic diagnosis of four RAG groups. Symbols represent individual patients. **c** Infections, autoimmunity, and immune dysregulation in RAG cohort (number of total cases). Inf, infection; ID, immune dysregulation; AI, autoimmune manifestation. **d** Clinical manifestations in RAG cohort distributed among the four groups (frequency as % total cases). Other: Miller-Fisher syndrome, tubulointerstitial nephritis, alopecia, vitiligo. PID-6 prenatal diagnosis. PID-7 No available data during first months of life

The cohort characteristics are described in Table 1. The majority of patients (8 cases, 36.4%) were affected by SCID presenting with T-B-NK+ phenotype except for two patients with T+B-NK+ phenotype in which maternal T engraftment was observed (PID-5 and PID-8), followed by OS (6 cases, 27.2%), LS/AS (4 cases, 18.2%), and CID (4 cases, 18.2%) (Fig. 1A, Tables 1 and 2). The age at presentation ranged from birth to 72 months, with a median of 5 months (range birth to 6 years), whereas the median age of genetic diagnosis was 6 months (range birth to 11 years).

In particular, the mean age of first symptom was 3.4 months (± 0.25 sd) and confirming genetic diagnosis 4.6 months (± 0.29 sd) for SCID, 2 months (± 0.19 sd) and 3 months (± 0.21 sd) for OS, 1 year (± 0.55 sd) and 1.2 years (± 0.53 sd) for LS/AS, and 3.2 years (± 2.06 sd) and 6.7 years (± 4.7 sd) for CID (Fig. 1B). The number of *RAG* diagnoses following NGS application changed from a median of 1.5 up to 2.6 per year (Fig. 1C). Noteworthy, there was no significant difference between patients with SCID and OS when comparing age at first clinical symptoms and at diagnosis (Fig. 1B). The median age of the first symptom and the genetic diagnosis were markedly lower in SCID and OS than in LS/AS and mostly in CID patients (Fig. 1B).

The symptom at presentation was infection (*n* = 18; 81.8%). Dermatitis represented the onset manifestation in all OS, two LS/AS (PID-15, PID-16) and two SCID patients (PID-5, PID-8). On the other hand, infections, autoimmunity, and immune dysregulation occurred together in most cases (*n* = 12; 54.5%). Two SCID and one OS early diagnosed due to prenatal diagnosis or strongly suggestive phenotype were promptly transplanted and did not present any severe manifestation (Fig. 1D). Chronic viremia was common among the cohort (SCID *n* = 4; OS *n* = 1; LS/As *n* = 3; CID *n* = 4), whereas respiratory and gastrointestinal infections were prevalent in SCID patients (Fig. 1E). Interestingly, 15 out of 22 (68.2%) patients presented autoimmune and/or hyperinflammatory manifestations (Fig. 1D). The most frequent was dermatitis occurring in 9 patients (40.9%). Lymphoproliferation and splenomegaly were present in 4 patients (18.1%). Nephropathy occurred in 2 patients (PID-14 and PID-16). whereas one patient developed Miller Fisher syndrome (PID-17) (Fig. S2, and Table 1).

**Fig. 2:**
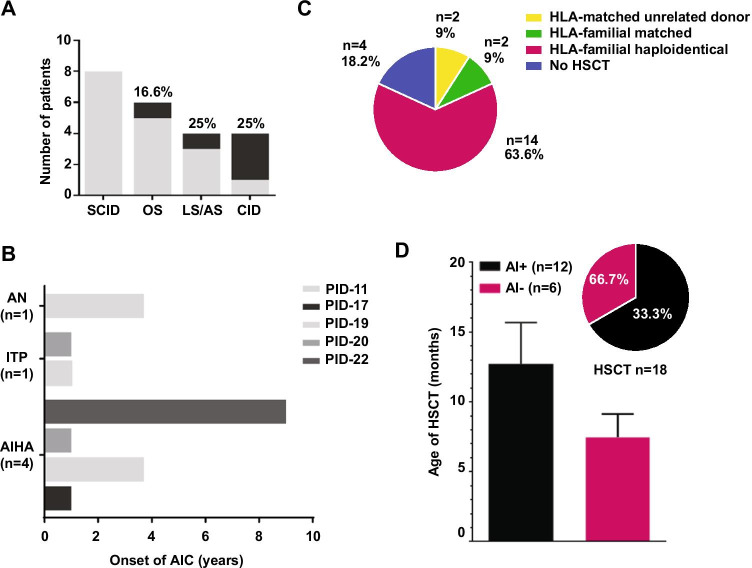
**a** Occurrence of autoimmune cytopenia in each RAG group. **b** Onset of AIC manifestation for PID-11,17,19,20,22. Autoimmune cytopenia (AIC), autoimmune neutropenia (AN), immune thrombocytopenia (ITP), autoimmune hemolytic anemia (AIHA). **c** Number of RAG diagnosis from 2009 to 2020. Pink background indicates the application of NGS. **c** Occurrence of HSCT in our RAG cohort. For each category, the total number of patients is indicated. **d** Occurrence of HSCT in patients with (AI + *n* = 12) or without (AI − *n* = 6) autoimmunity (frequency as % annotated total cases. *n* = 18) and age of HSCT

Notably, one OS, one LS/AS, and 3 CID patients (PID-12, PID-13, PID-17, PID-20, PID-10) with autoimmune/inflammatory complications experienced a severe autoimmune cytopenia (AIC) (Fig. 2A) with a median age at onset of 1 year (range 1–9) (Fig. 2B).

*RAG1* mutations were detected in nineteen (86.4%) patients, while *RAG2* mutations in three (13.6%) of them (Table S1). Consanguinity was documented in 9 out of 20 families (45%), in which we found homozygous mutations. We found three novel mutations in *RAG1* gene (p.S330LfsTer15, p.R404W, and p.N803S) and two in *RAG2* (p.H94R and p.N364VfsTer79). Combined Annotation-Dependent Depletion (CADD) scores have been used to define the degree of pathogenicity of novel mutations (Table S1).

### Immunological Phenotypes

As expected, increased IgE levels were observed in OS group. IgA and IgM resulted very low or undetectable in SCID and OS patients, whereas detected serum IgG in two SCID and three OS reflected maternal trans-placental transfer. Leaky/atypical SCID and CID patients showed normal immunoglobulin values except for one patient with LS/AS, PID-17, who manifested hypergammaglobulinemia (Fig. S1A and Table 2).

Total lymphocyte counts were severely reduced or almost lacking in SCID group (Table 2).

T cells were particularly low in SCID group, whereas CD3, CD4, and CD8 cell count were higher in OS patients. LS/AS and CID patients presented detectable T cells, albeit reduced proportion of naïve T cells (Fig. S1B-C). Expansion of T-cell receptor γδ was documented in two LS/AS patients (PID-16 and PID-17) (Fig. S1D), particularly following disseminated CMV infection as for PID-17. Circulating B cells were variably detected in AS and CID groups and were nearly absent as expected in SCID and OS patients correlating to their immunoglobulin levels. NK cells were present in all groups with normal or increased numbers (Fig. S1B).

### Outcomes and Treatment

All patients with AIC received high dose of intravenous immunoglobulins (IVIG) and steroids as first-line therapy with limited response. No complete remission was observed in any patients despite second-line therapies with Rituximab (PID-12, PID-17, PID-20), mycophenolate mofetil (MMF) (PID-20), rapamycin (PID-22), or third-line therapies with plasmapheresis (PID-17, PID-20).

Eighteen out of 22 patients underwent allogenic HSCT with a median of 7 months (range 3–36.7) (Fig. 2C). Donors were either matched unrelated (2 cases, with one cord blood and one bone marrow as stem cell source), HLA-phenotypically identical (2 cases transplanted from the mother), or HLA-haploidentical (14 patients). Conditioning regimen was based on Treosulfan 42 gr/m^2^, fludarabine 160 mg/m^2^, and ATLG, with 6 patients receiving additional tiotepa 10 mg/kg.

Neutrophils and platelets engraftment were achieved in 16 out of 18 patients after a median time of 15 days and 11 days, respectively. Two patients experienced primary graft failure and were rescued with an allograft from the other HLA-haploidentical parent.

Cumulative incidence of grades I–II acute graft-versus-host disease (aGvHD) was 17% (95% CI) with 3 patients experiencing mild, skin-only aGvHD. No cases of grades III–IV aGvHD were observed, and no patient experienced chronic GvHD. Infectious complications were frequent after HSCT, accounting for almost 90% of patients, and included blood stream infections (5 patients), viral reactivations (CMV, HHV6, and Adenovirus in 6, 2, and 4 patients, respectively), infectious enteritis (Rotavirus 1, Norovirus 1, and C. difficile 1 case), and invasive pulmonary aspergillosis (PID-19).

With a median follow-up of 61 months (range 17–138) overall survival of transplanted patients was 94% (95% CI). One patient died of massive pulmonary hemorrhage caused by invasive aspergillosis which was already present at the time of HSCT. Noteworthy, two additional patients of our cohort died before HSCT due to multiorgan failure.

Notably, all patients reached normal immunoglobulin levels.

Among fifteen patients with autoimmune/hyperinflammatory manifestations, HSCT was required for definitive management in twelve patients (80%). Patients with immune dysregulation underwent HSCT at an older age compared to those without immune dysregulation (median 12.7 vs. 7.4 years) partially due to the diagnostic delay (Fig. 2D). Two patients resolved treatment-refractory AIC, thanks to HSCT.

## Discussion

During an 11-year period, we diagnosed 22 RAG patients at the Bambino Gesù Children’s Hospital. The patients were classified as SCID, OS, LS/AS, and CID, confirming that different clinical phenotypes can be found in patients with identical mutations even within the same family.

We recorded an increased number of *RAG* diagnosis following NGS application from a median of 1.5 up to 2.6 per year. This was particular evident in the first year of NGS application, in which we unmasked previous undiagnosed cases. This increased frequency is consistent with recent reports, including newborn screening that confirm *RAG1/2* as the second most prevalent genes associated with SCID and the most common genes associated with leaky SCID/CID [29]. In addition, we observed a median delay of 75 days between clinical onset and genetic diagnosis for SCID, 1 month for OS, 14 months for LS/AS, and 3 years for CID patients. As reported in other studies [22], the CID diagnostic delay reflects a lack of recognition of hypomorphic RAG mutations associated with milder and late onset phenotype in contrast to the severe SCID phenotype caused by null RAG mutations. These data confirmed a higher frequency of RAG defects that should be promptly suspected and recognized to avoid severe complications.

As reported [30, 31], the immunological phenotype in our cohort confirmed the reduction of the T cell count and the diminished proportion of peripheral naive CD4 T cells in each group. Thus, a detailed immunophenotype could help in suspecting RAG deficiency in those patients with immune dysregulation.

*RAG1* mutations have been reported more frequently than *RAG2* [31, 32] as occurred in our cohort. Interestingly, we found three novel mutations in the *RAG1* gene and two in *RAG2*. Based on previously reported literature [33–35] that showed a significantly lower activity level of missense mutations in the nonamer-binding region (NBR) that is part of the catalytic core, we can assume that the p.R404W could explain the SCID phenotype observed in PID-8.

Similarly, the p.S330LfsTer15 mutation is found in PID-4 falls in the RING domain of RAG1, determining the absence of recombination activity by affecting protein subcellular localization or decreasing chromatin accessibility of the RAG complex [34, 35]; however, in this patient, the resulting truncated protein is the most evident cause of SCID phenotype. Moreover, the C-terminus of RAG1 protein has been identified primarily in patients with CID showing significant residual VDJ recombination activity [6]. These data perfectly reflect the p.N803S mutation carried by PID-22 characterized by a CID phenotype, although the recombination activity of this novel mutation was not yet determined. Finally, PID-12 carried the two novel p.H94R and p.N364VfsTer79 mutations, both in the extended catalytic core of the RAG2 protein that may affect its activity leading to an OS phenotype [36]. Also in this case, the p.N364VfsTer79 mutation causing an evident defective protein can more heavily affect the RAG2 function. Differently from other studies focused on populations with a high rate of consanguinity [37, 38], many patients enrolled in this work had non-consanguineous parents (59%), suggesting a high mutation rate in these genes as well as a large occurrence of heterozygous carriers.

Interestingly, although individuals with *RAG* heterozygous missense mutations were not reported to be typically associated to a RAG phenotype, we observed three CID patients presenting with autoimmune cytopenia and carrying only a single missense heterozygous mutation in *RAG* genes (data not shown). These data suggest that other factors, not yet fully understood, could influence the disease expression.

PID-16 and PID-20, defined as LS/AS and CID, respectively, carried the same homozygous mutations (p.R841W), with severe autoimmune manifestations: the second one, despite having received a timely diagnosis, died before being able to undergo HSCT. Similarly, two compound heterozygous siblings (PID-6 and PID-19) (p.R405G and p.R624H) were diagnosed as SCID and CID, respectively. The older sister (PID-19) diagnosed at 3 years of life died of invasive aspergillosis already present at time of HSCT. The younger sister (PID-6) who received at birth a more severe diagnosis of SCID underwent HSCT at 3 months of life showing a different clinical outcome, thanks also to a prenatal diagnosis. This underlines the importance of other epigenetic or environmental factors on disease course and how a prompt HSCT may be crucial.

Furthermore, four compound heterozygous *RAG1* patients, classified as SCID (PID-6), OS (PID-9), and CID (PID-19, PID-21), shared the same p.R624H mutation, with almost absent recombination activity, in combination with the p.R405G, p.Y728H, and p.R561H respectively. The p.Y728H showed a markedly decreased but detectable recombination activity in line with his milder phenotype [39], whereas p.R561H showed nearly absent activity (2.0 ± 0.6) reflecting the OS disease. Unfortunately, the p.R405G recombination activity was not still determined. Otherwise, the two compound heterozygous brothers (PID-11 and PID-14) with the p.R624C and the p.R841W mutations presented with the same OS disease. Thus, genotype–phenotype correlation in RAG deficiency is not absolute. Indeed, other factors such as chronic and uncontrolled infections may influence the outcome of the disease besides the level of recombination activity determining a variable clinical phenotype even within the same family. CMV, EBV, and HHV-6 viral infections probably affected the clinical course and the onset of complications in PID-19. On the contrary, we cannot know what the clinical course would have been in PID-6 if she had encountered a virus before HSCT.

Indeed, chronic, uncontrolled Herpesviridae viremia was frequent among CID group (75%). Usually, CID patients develop more severe manifestations often triggered by viral infections that closely preceded the onset of autoimmunity as in our patient PID-17 [40].

Nonetheless, the presence of residual B cells in CID patients makes them prone to EBV replication with higher risk of EBV-driven lymphoproliferation suggesting the need of a closer viral and radiological monitoring [41, 42].

PID-21 was initially classified as a humoral defect and only later reconsidered as CID at 11 years of age. Thus, considering the high frequency of hypomorphic mutations with a milder phenotype, a longitudinal re-evaluation of patients lacking a molecular diagnosis is always recommended, and RAG deficiency should also be suspected in patients where B cell defect is predominant. As rarely reported for other patients with CID phenotype [43], this patient did not undergo HSCT considering his good clinical status and the unsuccessful research for a suitable identical HSCT donor. He presented recurrent low respiratory infections well controlled by antibiotic prophylaxis (azithromycin) and immunoglobulin replacement therapy. This case underlines how the molecular definition could support pediatricians and immunologists in achieving better follow-up but open crucial questions on therapeutical options. Thus, a close follow-up in large multicentric international cohorts is mandatory to deepening the knowledge and understanding of these patients’ natural history. 

In line with other reports [22, 32], our data showed that severe immune dysregulation manifestations (68.2%) are a common feature of RAG deficiency often refractory to conventional medical management. In particular, dermatitis and autoimmune refractory cytopenia resulted in the most frequent manifestations. Otherwise, nephritis [22, 32], or neurological involvement (Miller-Fisher syndrome) could represent the first or the main symptom of onset [30], which can lead patients to the attention of other specialist delaying the correct diagnosis. The early recognition of these manifestations could contribute to the choice of a prompt specific treatment and prevent the onset of others complications [43, 44].

According to the literature [22], given the refractory nature of autoimmune cytopenia, it is essential to rapidly proceed to transplant patients with AIC to avoid complications or chronic infections that can be responsible for the onset of comorbidities compromising the outcome of HSCT. HLA-identical donor transplant demonstrates an excellent overall survival greater than 75% [45, 46]. Moreover, innovative HSCT approaches have shown encouraging results in case when an HLA-matched donor is not available [47, 48]. Finally, immunosuppressive or immunomodulatory drugs should be strongly considered to control immunodysregulation in these patients. 

## Conclusions

RAG deficiency still presents a challenge in tracing effective management and follow-up, considering the inability to predict the disease course in atypical cases. This study describes the clinical, immunological, and molecular characteristics of 22 RAG patients providing five novel mutations. This cohort highlighted the heterogeneity of manifestations associated with inborn error of immunity particularly for patients with hypomorphic mutations and milder phenotypes. In this regard, extending RAG analysis to older pediatric and adult patients affected by undefined CVID and/or immune dysregulation [42] might be helpful in discovering novel cases and expanding the current knowledge of natural history of *RAG* deficiency.

## Supplementary Information

Below is the link to the electronic supplementary material.

Supplementary file1 (DOCX 15 kb)

Supplementary file2 (DOCX 32.2 kb)

Supplementary Fig. 1 **Panel a** Immunoglobulin titers of each RAG group. Grey background indicates normal serum Ig values of normal donors. IgG. IgM. IgA normal donors ranges from [49] and IgE from Bambino Gesù Children’s Hospital. **Panel b** Immunophenotype shows absolute count of CD3+, CD4+, CD8+ T cell, CD19+ B cells and NK cells among groups. Panel c CD4+ CD45RA+ and CD4+CD45RO+ T-cell subsets, shown in LS/AS and CID. Panel d Percentage of TCRαβ and γδ T cells of LS/AS and CID. **P < 0.005. Shown are mean values ± SEM (PNG 509 kb)

High Resolution Image (TIF 1934 kb)

Supplementary Fig. 2 **Panel a** Abdominal CT scan and ultrasound showed hepatosplenomegaly in two CID patients. **Panel b** PID-17 magnetic resonance images showed marked thickening. impregnation of the roots of the cauda (left) and cranial nerves (right) reflecting a clinical picture of Miller Fisher’s Syndrome characterized by severe axial hypotonus. ophthalmoparesis. ataxia. generalized areflexia. progressive paralysis of cranial nerves. (PNG 619 kb)

High Resolution Image (TIF 2525 kb)

## Data Availability

Not applicable.

## Abbreviations

TCR: T-cell receptor
SCID: Severe combined immunodeficiency
OS: Omenn syndrome
AS: Atypical SCID
LS: Leaky/SCID
CID/G/A: Combined immunodeficiency with diffuse granulomatous disease and/or autoimmunity
EBV: Epstein-Barr virus
CVID: Common variable immunodeficiency
ICL: Idiopathic CD4 + T cell lymphopenia
NGS: Next generation sequencing
RTE: Recent thymic emigrants
IUIS: Union of immunological societies
PIDTC: Primary immune deficiency treatment consortium
CMV: Cytomegalovirus
IVIG: Intravenous immunoglobulin
ITP: Immune thrombocytopenia
AIHA: Autoimmune hemolytic anemia
HSCT: Hematopoietic stem cell transplantation
MAV: Myeloablative conditioning
MMF: Mycophenolate mofetil
aGvHD: Acute graft-versus-host disease
