# Supplementary material for: Clinical, Immunological, and Molecular Variability of RAG Deficiency: A Retrospective Analysis of 22 RAG Patients
Source: J Clin Immunol. 2021 Oct 18;42(1):130–45. doi: 10.1007/s10875-021-01130-3 (PMC8821501; doi:10.1007/s10875-021-01130-3)
Supplement: Supplementary file 1 — Supplementary file1 (DOCX 15 kb) [file 10875_2021_1130_MOESM1_ESM.docx]

**Particular LS/CID cases**

Three patients need a more in-depth description because of the peculiarities of clinical phenotype. In details, PID-17 carried a homozygous frameshift mutation and experienced a persistent CMV viremia, *P. aeruginosa* sepsis and a mild BCGitis at the age of seven months. In addition, he developed Miller Fisher’s Syndrome (Fig. S2a, b). Immunological evaluation revealed marked CD4-penia, expansion of γδ+ T-cells and memory B cells with hypergammaglobulinemia and ITP. In order to control the expansion of B autoreactive clones, Rituximab therapy and plasmapheresis were used allowing a complete depletion of B cells and progressive improvement of neurological and respiratory function. Patient received HLA-haploidentical transplant from the father with full engraftment. Four months post-transplantation severe AIHA associated to a CMV reactivation was observed. BAFF plasma level resulted increased (12.000 pg/ml) as previously described in patients with RAG mutations and autoimmunity [1-3]. Antiviral drugs, high-dose IVIg, Rituximab, plasmapheresis and multiple blood transfusions were required to control autoimmunity and achieve a complete remission.

PID-16 carrying the homozygous p.R841W *RAG1* mutation was characterized by long lasting diarrhea, erythroderma, candidiasis and tubulo-interstitial nephritis with infiltrate of lymphocytes, histiocytes, plasmacells and eosinophils. He showed T+B-NK+ phenotype due to maternal T engraftment. Immunological evaluation revealed CD4-, CD8-penia and γδ+ T-cells expansion.

Lastly, PID-21, previously reported [39], presented a disease onset at six years of age characterized by relapsing nasal polyposis, severe agammaglobulinemia and absence of B cell suggesting initially a humoral defect. His past history was notable for recurrent middle-ear infections, chickenpox and mild persistent EBV viremia. Bone marrow examination showed a marked decrease of B cell progenitors, with an incomplete arrest at pro-B cell stage and few pre-B and mature B cells. No sign of myelodysplasia was found. Later in the follow-up a marked reduction of CD4+/CD45RA+/CD31+ recent thymic emigrants (RTE) were detected over-time, along with a corresponding increase in the proportion of memory T cells. Since T lymphocytes pool deteriorate with age, we reassessed his clinical diagnosis that changed from agammaglobulinemia to CID. This patient, who did not receive HSCT, is currently alive and healthy on IVIGs replacement therapy at the age of 16 years.

**REFERENCES**

1. Cassani B, Poliani PL, Marella V, Schena F, Sauer AV, Ravanini M, et al. Homeostatic expansion of autoreactive immunoglobulin-secreting cells in the Rag2 mouse model of Omenn syndrome. J Exp Med. 2010; 207:1525–1540. [PubMed: 20547828]
2. Walter JE, Rucci F, Patrizi L, Recher M, Regenass S, Paganini T, et al. Expansion of immunoglobulin-secreting cells and defects in B cell tolerance in Rag-dependent immunodeficiency. J Exp Med. 2010; 207:1541–1554. 82. Lesley R, et al. Reduced competitiveness of autoantigen-engaged B cells due to increased dependence on BAFF. Immunity. 2004; 20:441–453. [PubMed: 15084273]
3. Thien M, Phan TG, Gardam S, Amesbury M, Basten A, Mackay F et al. Excess BAFF rescues self-reactive B cells from peripheral deletion and allows them to enter forbidden follicular and marginal zone niches. Immunity. 2004; 20:785–798. [PubMed: 15189742]
