# Supplementary material for: Clinical, Immunological, and Molecular Variability of RAG Deficiency: A Retrospective Analysis of 22 RAG Patients
Source: J Clin Immunol. 2021 Oct 18;42(1):130–45. doi: 10.1007/s10875-021-01130-3 (PMC8821501; doi:10.1007/s10875-021-01130-3)
Supplement: Supplementary file 2 — Supplementary file2 (DOCX 32.2 kb) [file 10875_2021_1130_MOESM2_ESM.docx]

**Supplementary Table 1** Molecular Characterization of RAG patients. SCID, Severe Combined Immunodeficiency; OS, Omenn Syndrome; AS, Atypical SCID; LS, Leaky/SCID; CID, Combined immunodeficiency; dbSNP, Single Nucleotide Polymorphism Database; CADD, Combined Annotation Dependent Depletion; MAF, Minor Allele Frequency. Bold: novel not described mutations. **Values from reference 51. Frequency and CADD scores were reported only for novel variants when available. n.d. not determined.

| **ID** | **Disease** | **Consanguinity/**  **Country** | **Gene** | **Mutation** | | **RefSNP; frequency (gnomAD exomes) and CADD score** | **Zygosity** | **Inheritance** | **Protein Domain** | **Recombinase activity allele a (approximately)** | **Recombinase activity allele b (approximately)** | **Ref** |
| --- | --- | --- | --- | --- | --- | --- | --- | --- | --- | --- | --- | --- |
| PID-1 | SCID | No/  Italy  (Lazio) | *RAG2* | a) c.1A>G; p.M1V | b) c.1403_1406del; p.H468RfsTer16 | a) rs1564997814  b) rs786205616 | Compound heterozygous | Familial | a) core region | n.d. | 0% deduced | [52] |
| PID-2 | SCID | No/  Italy  (Lazio) | *RAG1* | a1) c.1681C>T; p.R561C  a2) c.1815G>C; p.M605I | b) c.2780T>C; p.F927S | a1) rs104894285;  a2) rs1564989420  b) rs1249862287 | Compound heterozygous | Familial | a1) pre RNAseH  (catalytic core);  a2) catalytic RNAaseH (catalytic core)  b) b) NBR (catalytic core) | n.d.; n.d. | n.d. | [52] |
| PID-3 | SCID | No/  Italy (Campania) | *RAG1* | a) c.1229G>A; p.R410Q | b) c.1863delG; p.A622QfsTer9 | a) rs199474684;  b) rs1564989455 | Compound heterozygous | n.d. | a) NBR (catalytic core) | 0% | 0% deduced | [52] |
| PID-4 | SCID | Yes/  Lebanon | *RAG1* | **a) c.987delC;** **p.S330LfsTer15** |  | a) rs1564988767  f: - | Homozygous | n.d. probably familial | a) RING domain  (no-core) | 0% deduced |  | [52] |
| PID-5 | SCID | No/  Italy  (Roma) | *RAG1* | a) c.1228C>T; p.R410W | b) c.2780T>C; p.F927S | a) rs758288006  b) rs1249862287 | Compound heterozygous | Familial | a) NBR (catalytic core) b) Zn binding domain (catalytic core) | 0.0 ± 0.0** | n.d. |  |
| PID-6 | SCID | No/  Italy  (Marche) | *RAG1* | a) c.1871G>A; p.R624H | b) c.1213A>G; p.R405G | a) rs199474680  b) rs1564988958 | Compound heterozygous | Familial | a) catalytic RNAaseH (catalytic core) b) NBR (catalytic core) | 0.0 ± 0.4** | n.d. |  |
| PID-7 | SCID | Yes/  Syria | *RAG1* | a) c.1361 T>A p. L454Q |  | a) rs199474677 | Homozygous | n.d. probably familial | a) NBR (catalytic core) | 5.4 ± 0.7** |  |  |
| PID-8 | SCID | Yes/  Lebanon | *RAG1* | a) **c. 1210 C>T; p.R404W** |  | a) rs764981110  f: 0.00000399  CADD: 25 | Homozygous | n.d. probably familial | a) NBR (catalytic core) | n.d. |  |  |
| PID-9 | OS | No/  Italy  (Puglia) | *RAG1* | a) c.1682G>A; p.R561H | b) c.1871G>A; p.R624H | a) rs104894284  b) rs199474680 | Compound heterozygous | Familial | a) pre RNAseH  (catalytic core);  b) catalytic RNAaseH (catalytic core) | 2.0 ± 0.6** | 0.0 ± 0.4** | [52] |
| PID-10 | OS | No/  Italy  (Campania) | *RAG1* | a)c.351delT; p.Phe118LeufsTer21 | b) c.1577T>G; p.L526R | a) rs1480764714  b) – | Compound heterozygous | n.d. | a) pre RNAseH  (catalytic core); | 0% deduced | n.d. |  |
| PID-11 | OS | No/  Italy  (Lazio) | *RAG1* | a) c.1870C>T; p.R624C | b) c.2521C>T; p.R841W | a) rs199474688  b) rs104894287 | Compound heterozygous | Familial | a) catalytic RNAaseH (catalytic core) b) Zn binding domain (catalytic core) | n.d. | 10.0 ± 0.5** | [52] |
| PID-12 | OS | No/  Italy  (Lazio) | *RAG2* | **a) c.281A>G; p.H94R** | **b) c.1090_1093delAACA;** **p.N364VfsTer79** | a) -  f: -  CADD 25.3  b) rs756470858  f: 0.00000398 | Compound heterozygous | Familial | a) core domain  b) core domain | n.d. | n.d. |  |
| PID-13 | OS | Yes/  Tunisia | *RAG1* | a) c.519delT; p.E174Sfs*27 |  | a) rs1241698978 | Homozygous | n.d. probably familial | a) CND (central non-core domain) term | 0.5 ± 0.2** |  |  |
| PID-14 | OS | No/  Italy  (Lazio) | *RAG1* | a) c.1870C>T; p.R624C | b) c.2521C>T; p.R841W | a) rs199474688  b) rs104894287 | Compound heterozygous | Familial | a) catalytic RNAaseH (catalytic core) b) Zn binding domain (catalytic core) | n.d. | 10.0 ± 0.5** |  |
| PID-15 | LS/AS | Yes/  Lebanon | *RAG2* | a) c.685C>T; p.R229W |  | a) rs765298019 | Homozygous | n.d. probably familial | a) core region | 10.5 ± 0.5** |  | [52] |
| PID-16 | LS/AS | Yes/  Lebanon | *RAG1* | a) c.2521C>T; p.R841W |  | a) rs104894287 | Homozygous | Familial | a) Zn binding domain (catalytic core) | 10.0 ± 0.5** |  | [52] |
| PID-17 | LS/AS | Yes/  North  Macedonia | *RAG1* | a) c.256_257del; p.K86VfsTer33 |  | a) rs772962160 | Homozygous | Familial | a) N-term | 2.7 ± 0.3** |  | [51] |
| PID-18 | LS/AS | Yes/  Lebanon | *RAG1* | a) c.1767C>G; p.Y589X |  | a) rs991089005 | Homozygous | n.d. proably familial | a) core region | 0% deduced |  |  |
| PID-19 | CID | No/  Italy  (Marche) | *RAG1* | a) c. 1871G>A; p.R624H | b) c.1213A>G; p.R405G | a) rs199474680  b) rs1564988958 | Compound heterozygous | Familial | a) catalytic RNAaseH (catalytic core) b) NBR (catalytic core) | 0.0 ± 0.4** | Recombinase activity ongoing | [52] |
| PID-20 | CID | Yes/  Ecuador | *RAG1* | a) c.2521C>T; p.R841W |  | rs104894287 | Homozygous | Familial | a) Zn binding domain (catalytic core) | 10.0 ± 0.5** |  | [52] |
| PID-21 | CID | No/  Italy | *RAG1* | a) c.1871G>A; p.R624H | b) c.2182T>C; p.Y728H | a) rs199474680  b) rs1564989655 | Compound heterozygous | Familial | a) catalytic RNAaseH (catalytic core) b) Zn binding domain (catalytic core) | 0.0 ± 0.4** | 25.7% | [39] |
| PID-22 | CID | No/  Sri Lanka | *RAG1* | a) c.2095C>T; p.R699W | **b) c.2408A>G; p.N803S** | a) rs199474676  b) -  f: -  CADD 24.7 | Compound heterozygous | n.d. | a) catalytic RNAaseH (catalytic core) b) Zn binding domain (catalytic core) | 19.3 ± 1.8** | n.d. |  |

**Reference**

51. Lawless D, Lango Allen H, Thaventhiran J, Anwar R, Fellay J, Walter JE, Savic S, NIHR BioResource–Rare Diseases Consortium. Predicting the occurrence of variants in RAG1 and RAG2. J Clin Immunol. 2019;39(7):688–701. https://doi.org/10.1007/s10875-019-00670-z.
