# Supplementary figures and images for: Clinical, Immunological, and Molecular Variability of RAG Deficiency: A Retrospective Analysis of 22 RAG Patients
Source: J Clin Immunol. 2021 Oct 18;42(1):130–45. doi: 10.1007/s10875-021-01130-3 (PMC8821501; doi:10.1007/s10875-021-01130-3)

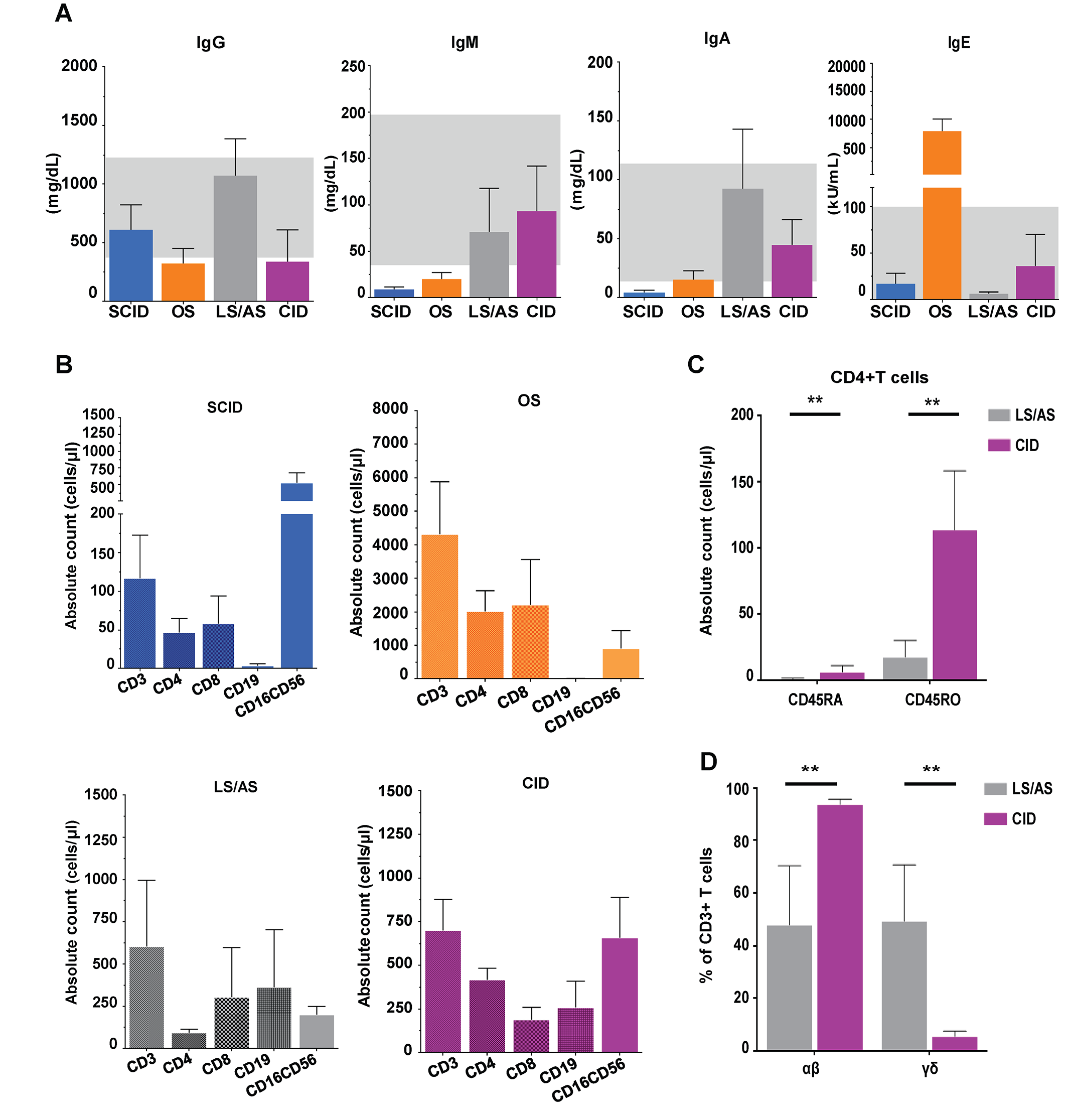

Supplement: Supplementary file 3 — Panel a Immunoglobulin titers of each RAG group. Grey background indicates normal serum Ig values of normal donors. IgG. IgM. IgA normal donors ranges from [49] and IgE from Bambino Gesù Children’s Hospital. Panel b Immunophenotype shows absolute count of CD3+, CD4+, CD8+ T cell, CD19+ B cells and NK cells among groups. Panel c CD4+ CD45RA+ and CD4+CD45RO+ T-cell subsets, shown in LS/AS and CID. Panel d Percentage of TCRαβ and γδ T cells of LS/AS and CID. **P < 0.005. Shown are mean values ± SEM (PNG 509 kb) [file 10875_2021_1130_Fig3_ESM.png]

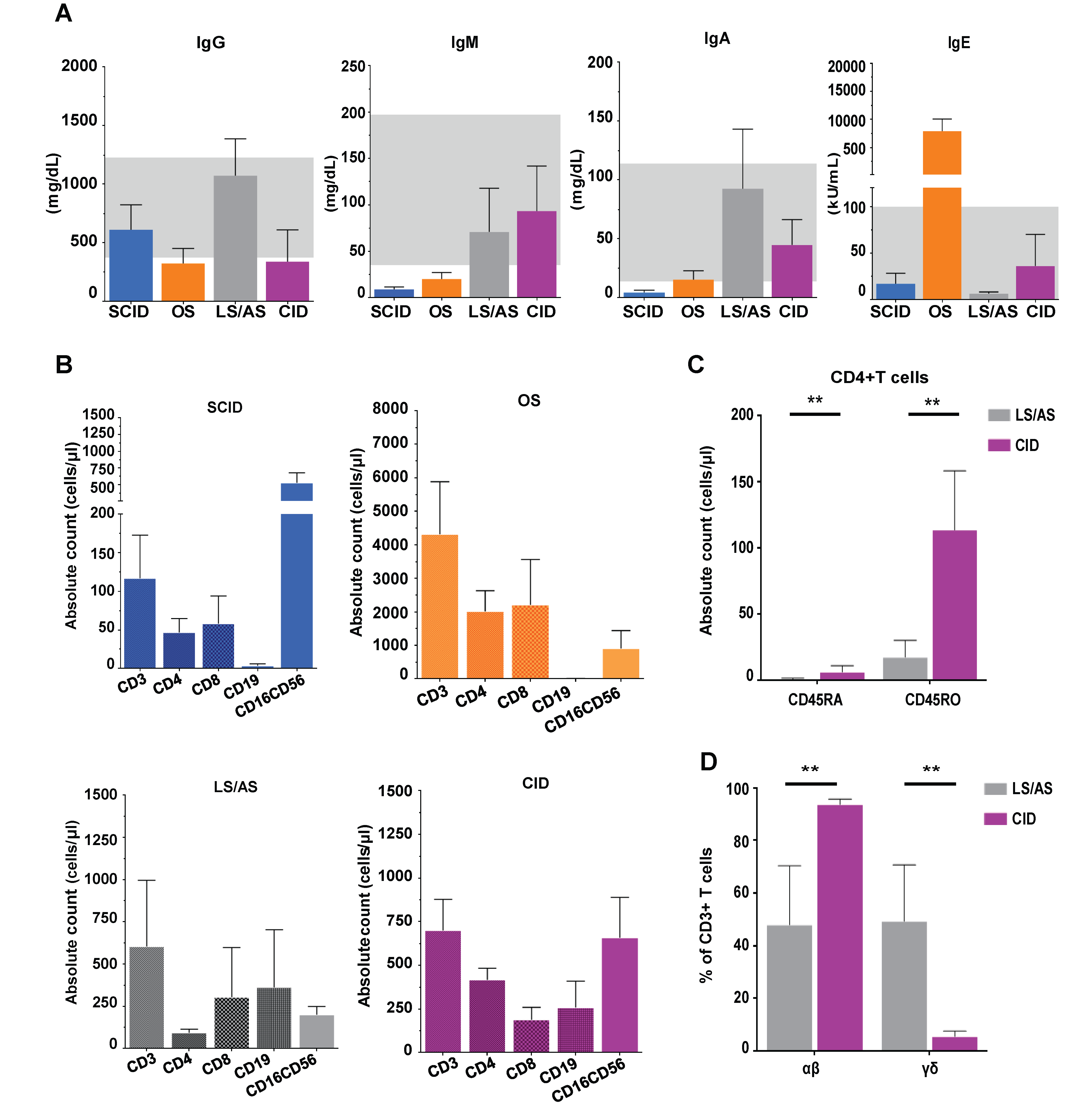

Supplement: Supplementary file 4 — High Resolution Image (TIF 1934 kb) [file 10875_2021_1130_MOESM3_ESM.tif]

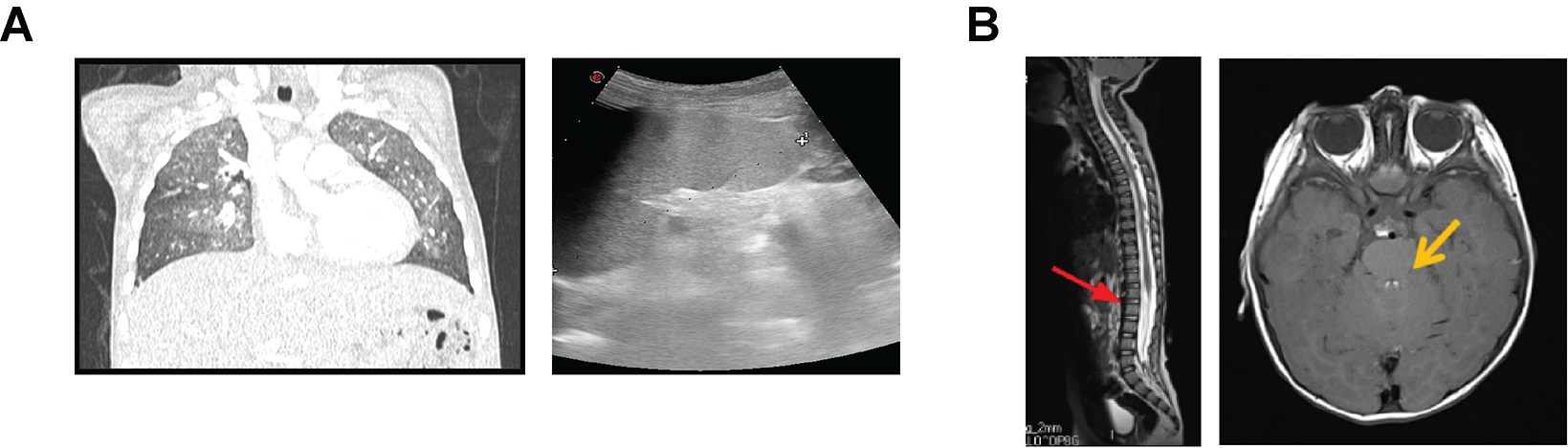

Supplement: Supplementary file 5 — Panel a Abdominal CT scan and ultrasound showed hepatosplenomegaly in two CID patients. Panel b PID-17 magnetic resonance images showed marked thickening. impregnation of the roots of the cauda (left) and cranial nerves (right) reflecting a clinical picture of Miller Fisher’s Syndrome characterized by severe axial hypotonus. ophthalmoparesis. ataxia. generalized areflexia. progressive paralysis of cranial nerves. (PNG 619 kb) [file 10875_2021_1130_Fig4_ESM.png]

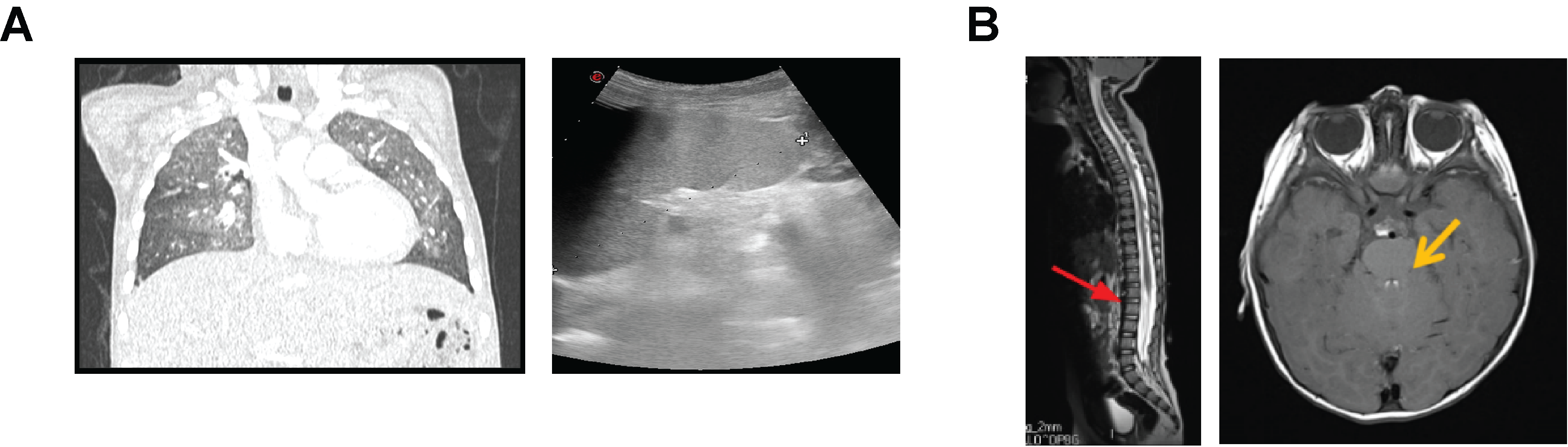

Supplement: Supplementary file 6 — High Resolution Image (TIF 2525 kb) [file 10875_2021_1130_MOESM4_ESM.tif]
